# Proton Beam Therapy for Advanced Periocular Skin Cancer: An Eye-Sparing Approach

**DOI:** 10.3390/cancers17020327

**Published:** 2025-01-20

**Authors:** Yingying Zhang, Isabela C. S. Lima, Alessandra A. Woo, Stephen Zieminski, Judith A. Adams, Megan A. Hughes, Annie W. Chan

**Affiliations:** 1 Department of Radiation Oncology, Massachusetts General Hospital, Harvard Medical School, 55 Fruit Street, Cox 308, Boston, MA 02114, USA; 2 Department of Oncology, Xiangya Hospital of Central South University, No. 87 Xiangya Road, Changsha 410078, China

**Keywords:** proton beam therapy, periocular skin cancer, orbital sparing, ocular sparing, eye sparing, Mohs surgery, orbital exenteration, squamous cell carcinoma, basal cell carcinoma

## Abstract

**Background/Objectives:** The management of periocular skin malignancies presents a unique challenge. Proton beam therapy, due to its sharp dose fall-off, allows for the delivery of a tumoricidal dose to the tumor while sparing adjacent normal tissues. **Methods:** Thirteen patients with a median age of 76.5 years received protons at our institution to a median dose of 66.6 Gy (RBE). Sixty-four percent of the lesions were basal cell carcinoma, and 22% were squamous cell carcinoma. Eighty-six percent of patients underwent biopsy only or partial resection. Fifty-seven percent of the lesions were located in the medial or lateral canthus. There was orbital invasion in 93% of the cases. Locoregional control probability and overall survival were estimated with the Kaplan–Meier method. Treatment toxicity was scored using the CTCAE 4.0. **Results:** At a median follow-up of 96 months, there was no local recurrence. The rate of orbital preservation was 100%. Functional vision was maintained in all the patients. There was no acute or late grade 3 or higher toxicity. **Conclusions:** Protons allow for long-term tumor control with eye preservation in patients with locally advanced periocular skin cancers. Larger prospective multi-institutional trials with standardized ophthalmological assessments are needed to confirm our findings.

## Simple Summary

The standard of care for the treatment of locally advanced periocular skin cancer is surgical resection, which commonly involves orbital exenteration, albeit normal vision in the majority of patients. The current study reports the long-term outcomes of using proton beam therapy as an eye-sparing approach in the treatment of locally advanced periocular skin cancer. Our findings suggest that proton beam therapy is an effective treatment, allowing tumor control while preserving functional vision in this group of patients.

## 1. Introduction

Skin cancer is the most common cancer in the United States [1] with one in five Americans will develop skin cancer in their lifetime [2]. Skin cancer frequently occurs in the periocular area due to chronic ultraviolet exposure. Basal cell carcinoma is the most common periorbital skin cancer, followed by squamous cell carcinoma [3].

Management of skin cancer in the periocular areas presents special challenges. Local excision with orbit preservation is considered the standard of care for small and superficial periocular skin cancer. However, for advanced tumors, orbital exenteration, which involves complete removal of periorbital tissues and orbital contents, is commonly performed in the presence of normal visual function and unproven survival benefit in this group of patients [4].

Radiation therapy plays an indispensable role in the management of periocular skin cancer, both in the definitive and postoperative settings. Though skin cancer is sensitive to radiation, the orbital anatomy and radiosensitivity of ocular structures provide unique challenges. Commonly used radiation modalities for periorbital skin cancer include intensity-modulated radiation therapy (IMRT), electrons, stereotactic radiosurgery, and brachytherapy. Though the local control rates are optimal with these techniques, ocular toxicities such as keratopathy, retinopathy, optic neuropathy, dry eye, cataracts, and optic neuritis resulting in chronic eye pain and vision decline are commonly unavoidable [5,6].

Proton beam therapy is an excellent option for periocular tumors. Its sharp lateral penumbra allows for the sparing of normal tissues, such as the cornea, whereas the zero-exist dose of the proton’s Bragg peak allows for the sparing of normal tissues such as the optic nerve and retina beyond the target. As the beam sharpness of protons decreases with depth, skin cancer with its superficial location represents one of the best indications for protons. The objective of this study is to evaluate the long-term outcomes in patients with periocular skin cancer treated with proton beam therapy.

## 2. Materials and Methods

Thirteen patients with 14 periorbital skin cancers were treated with proton beam therapy between 2006 and 2014 at the Massachusetts General Hospital. The study was approved by the institutional review board. To obtain long-term outcomes, patients who were treated in 2015 and after were excluded from the study. Patients who underwent orbital exenteration prior to protons were also excluded. All available pathology slides were reviewed at our institution before treatment. Each patient was evaluated by a multidisciplinary team including a radiation oncologist, a medical oncologist, an ophthalmologist, and a surgical oncologist. A collective decision was made for patients to undergo an ocular-sparing approach with proton beam therapy.

### 2.1. Surgery

The extent of surgery, including withholding orbital exenteration or eye enucleation, was made jointly by the multidisciplinary team. The extent of surgery varied by tumor location. Eighty-six percent of patients underwent only biopsy or partial resection.

### 2.2. Proton Planning and Delivery

All potential new patients were discussed at the departmental weekly proton rounds. Radiation oncologists, dosimetrists, and medical physicists attended to decide the radiation treatment after a thorough review of clinical history and imaging studies. Those cases would only be approved when protons could potentially result in improved dosimetric and clinical outcomes compared with photon therapy.

All patients underwent diagnostic computed tomography (CT) and magnetic resonance imaging (MRI) scans before making the treatment plan. A thin-cut (2.5 mm/slice) high-resolution CT scan with intravenous contrast medium was obtained with the patient immobilized in a customized thermoplastic mask. For each CT, Hounsfield units (HUs) were sampled to calibrate the conversion curve for HUs to proton stopping power (relative to water) [7].

High-resolution MRI with contrast was used to assist in delineating targets. T1-weighted non-contrast and contrast-enhanced MRI of the orbits were performed. For axial images, regions from the superior margin of the orbital rim to the inferior margin of the orbital rim, angled to be in-plane with the orbital segments of the optic nerves, were obtained. For coronal images, regions from the anterior margin of the globe to the anterior surface of the pons, perpendicular to the axis of the orbital segment of the optic nerves, were obtained. MRI of the skull base was also performed to evaluate perineural invasion in the skull base.

Gross tumor volume (GTV) and clinical target volumes (CTV) comprising the high-risk microscopic disease were manually contoured by the treating radiation oncologist. Corneas, retinas, lens, lacrimal glands, optic chiasm, optic nerves, lens, brain, brainstem, and other avoidance structures were delineated or verified by an experienced head and neck neuroanatomist for each patient.

All patients were treated with curative intent with 230 MeV protons by using a double passive scattering technique. Proton planning was accomplished using a non-commercial in-house modified XiO treatment planning system (CIO Inc, St Louis, MO, USA). An energy selection system, modulator wheels, range shifters, and range compensators were selected to spread out the proton Bragg peak and to reduce the proton range leading the Bragg peak to cover the whole tumor depth. Individual treatment fields were shaped by range compensators and apertures designed for each patient in the treatment-planning system. For each beam, a Lucite range compensator was created to achieve optimal distal shaping while sparing structures behind the targets. To spare the orbital structures such as the lens, cornea, and retina adjacent to the tumor, a brass aperture was applied to shape the beam laterally. Due to the narrow penumbra of protons at superficial tissues, a very steep lateral dose fall-off was achieved in superficial targets such as cutaneous malignancies.

### 2.3. Follow-Up and Assessment

During the proton radiation treatment, patients were examined weekly. A follow-up was performed approximately 2 to 6 weeks after treatment and then every 3 to 6 months thereafter. Patients received routine physical examination and imaging, including CT and MRI of the orbit. Comprehensive ocular examinations, including assessments of visual acuity, fundus examination, and corneal topography were performed by ophthalmologists. Acute radiation-related toxicity and late toxicity were recorded and graded according to the National Cancer Institute’s Common Terminology Criteria for Adverse Events version 4.0 (CTCAE v4.0). Late toxicities were defined as events occurring or persisting greater than 90 days after the start of proton treatment.

### 2.4. Statistical Analysis

The primary endpoint of the study was local control with orbital preservation. The secondary endpoint was treatment-related toxicity. The Kaplan–Meier method was used to estimate the local and nodal probabilities. Local and nodal recurrence was defined as the time from the first day of radiation to recurrence or last known follow-up without evidence of disease, censoring patients at the last follow-up or death.

## 3. Results

### 3.1. Clinical Features

Table 1 summarizes the patient, tumor, and treatment characteristics. There were 11 females and 3 males. The median age at the time of proton therapy was 76.5 years old. The median ECOG score was 1. Sixty-four percent of patients had basal cell carcinomas and 22% squamous cell carcinomas. The medial canthus and lower eyelid were the most common sites. Sixty-four percent of the cases were recurrent tumors after surgery. One patient initially had basal cell carcinoma at the right medial canthus in 2007 and was cured with protons; however, three years later, she was diagnosed with a new second basal cell carcinoma at the right lateral canthus. Orbital invasion (clinical or radiographic) was observed in 93% of the patients.

### 3.2. Multidisciplinary Treatment

Fifty percent of the cases underwent two or more surgical procedures because of repeated recurrences. Before proton therapy, six cases underwent one to four Mohs surgeries, five cases underwent one to two local excisions, and one underwent a local excision for the primary tumor and Mohs surgery for the recurrence. One case underwent cryotherapy followed by local excision and lateral orbitotomy for subsequent recurrences.

The mean time interval between surgery and the initiation of protons was 10.6 weeks. All patients received proton radiation to the gross tumor or primary tumor bed. None of the patients received nodal irradiation. The median proton dose to the GTV was 66.6 Gy(RBE) ([66–70 Gy(RBE)] and CTV was 60 Gy(RBE) [(54–66 Gy(RBE)]. One patient received concurrent weekly carboplatin.

### 3.3. Treatment Outcomes

The median follow-up time for all surviving patients was 96.0 months (range, 33–180 months). All patients had at least 2 years of follow-up after proton beam therapy. The local control rate was 100%. There was no nodal recurrence or distant metastasis. No patient required orbital exenteration or enucleation. Functional vision was maintained in all the patients.

### 3.4. Dosimetry

All patients were treated with the passive-scattering proton technique. Two representative cases are illustrated below.

#### 3.4.1. Case 1 (Figure 1)

**Figure 1:**
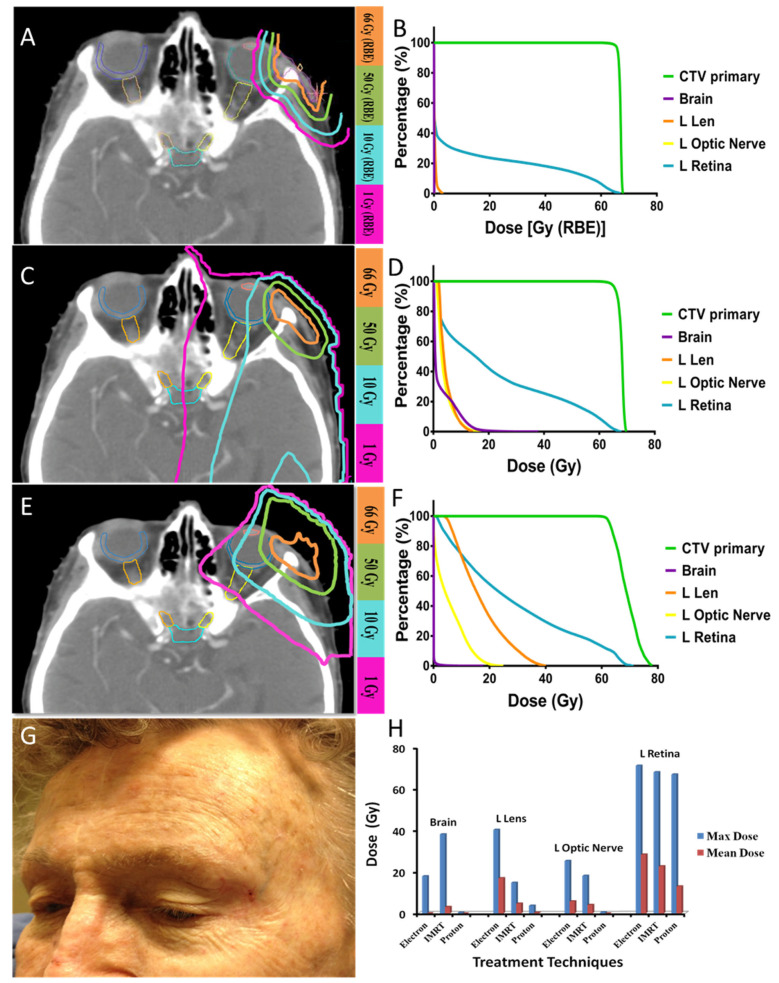
(**A**) Proton treatment plan. (**B**) Dose-volume histogram (DVH) of the proton plan in Figure 1A. (**C**) IMRT treatment plan. (**D**) DVH of the IMRT plan in Figure 1C. (**E**) Electron treatment plan. (**F**) DVH of the electron treatment plan in Figure 1E. (**G**) Picture of the treated area in the left lateral canthus taken 5 years after the completion of proton beam therapy. (**H**) Comparison of doses to the organs at risk with protons, IMRT, and electrons.

An 80-year-old female with squamous cell carcinoma on the left lateral canthus with the involvement of the lateral orbital rim, lacrimal gland, and lateral rectus muscle. A light thin pink line delineates the gross tumor at the time of proton beam therapy (Figure 1A). The patient had undergone a partial resection. Gross total resection would involve orbital exenteration of the normal-functioning eye. The patient underwent proton beam therapy to a total dose of 66 Gy(RBE) (Figure 1A,B). During a 12-year follow-up after protons, the tumor was under control with excellent visual and cosmetic outcomes. With the use of protons, the left lens and the left optic nerve received 1 Gy(RBE) or less, despite being only millimeters away from the tumor target. Figure 1C,D, and Figure 1E,F showed the six MV IMRT and six MeV electron plans of the same patient, respectively. Figure 1G shows the photograph taken 60 months after the completion of proton therapy. The patient was able to preserve her vision and did not develop any long-term side effects from protons other than a very mild skin telangiectasia in the irradiated area and loss of the lateral aspect of the left eyebrow. Figure 1H shows the comparison of doses to the organs at risk with protons, IMRT, and electron plans. Protons are less penetrating and exhibit less lateral scattering than electrons and IMRT. Protons result in significantly lower maximal and mean doses to all organs at risk when compared to electrons or IMRT.

#### 3.4.2. Case 2 (Figure 2)

**Figure 2:**
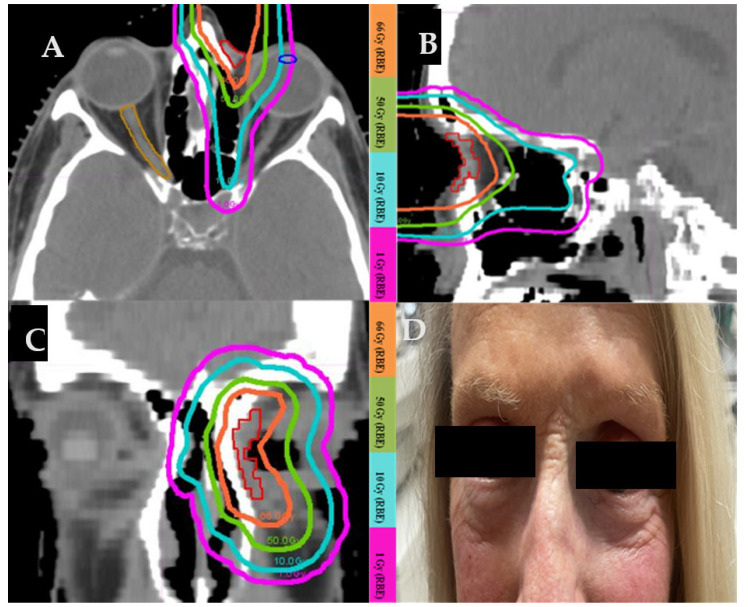
Proton treatment plan in axial (**A**), sagittal (**B**), and coronal (**C**) plane. (**D**) Picture of the treated area in the left medial canthus taken 14 years after the completion of proton therapy.

A 63-year-old female with biopsy-proven recurrent basal cell carcinoma on the left medial canthus with post-septal involvement. Surgery would require orbital exenteration of the normal functioning eye. She underwent definitive proton beam therapy with a total dose of 66 Gy(RBE). Figure 2A–C show the axial, sagittal, and coronal views of dose distribution of this patient’s proton plan. The photograph (Figure 2D) was taken 14 years after the completion of protons. There was no evidence of a recurrence. The patient developed mild skin hypopigmentation in the irradiated area and loss of the medial aspect of the left eyebrow.

### 3.5. Toxicity

For patients who received surgical procedures before protons, their treatment toxicities were still recorded as radiation-related events even though surgery might also influence the risk and extent of side effects. Generally, all patients tolerated proton treatment very well. No patients required a treatment break. There was no acute or late grade 3 or higher toxicity.

### 3.6. Acute Toxicity

Overall, the acute toxicity was limited. There was no new grade 3 or higher acute toxicity. Four patients developed grade 1 (relieved by rest) and one patient grade 2 (not relieved by rest, limiting activities of daily living) fatigue. As the skin was the target of radiation, all patients developed the expected moderate erythema (grade 2 radiation dermatitis) which was managed without difficulty. One patient developed a grade 1 (asymptomatic) nasal congestion. All acute ocular symptoms were grade 1 with no symptoms or interventions needed, including two patients with dry eyes, three patients with epiphora, and three patients with conjunctival injections.

### 3.7. Late Toxicity

There was no late grade 3 or higher toxicity. All patients were able to preserve their eyes with useful vision. One patient developed grade 1 loss of medial eyebrow. All other late effects were ocular events, including three patients with grade 1 epiphora, three patients with grade 1 dry eyes, three patients with grade 1 conjunctival telangiectasia, four patients with grade 1 ectropion, two patients with limited peripheral retinopathy, and one patient with grade 2 (symptomatic) exposure keratopathy. Two patients developed cataracts (grade 2) bilaterally, including the untreated eyes.

## 4. Discussion

With a median follow-up of 96.0 months, the treatment of primary and recurrent periocular skin malignancies with proton beam therapy at our institution resulted in a local control rate of 100% and limited acute and long-term side effects. Despite the invasion of orbital structures in the majority of patients, there was no acute or late grade 3 toxicity. The eye retention rate was 100%. Functional vision was maintained in all the patients.

Traditional eye-sparing radiation techniques for recurrent or inoperable periocular skin cancer include electrons, orthovoltage, brachytherapy, 3D-conformal radiation therapy, and intensity-modulated radiation therapy (IMRT) [8]. The local recurrence rates were reportedly approximately 10% or lower with these techniques [8,9,10,11,12]. Complications such as conjunctival scarring, epiphora, dry eye, ectropion, eyelid deformity, and keratitis were reported [9,10,11,12]. Superficial radiation such as electrons, orthovoltage, and brachytherapy have their limitations. For electrons and orthovoltage radiotherapy, the use of internal lead or tungsten protective eye shields during daily treatments, which are uncomfortable for patients, is necessary to protect the eyes. For patients who have undergone eyelid surgery, the placement of internal protective eye shields may not be feasible. Brachytherapy, which involves the insertion of radioactive catheters into the tumor bed, is an invasive procedure that only allows superficial tumors to be targeted. Three-dimensional conformal radiation therapy and IMRT, while allowing deeper tumors to be targeted, expose a large number of normal tissues to radiation.

Proton beam therapy offers an extremely attractive approach for periocular skin cancer. Its distinctive physical properties allow for significant sparing of normal tissues lateral and distal to the tumor targets. It is non-invasive and does not require the use of any eye shield. It also does not have any depth limitation. Proton beams have been used in the treatment of periorbital skin malignancies [13,14,15]. The University of Florida reported the outcome of 26 non-melanoma skin cancer patients with clinical perineural invasion [13]. At a median follow-up of 2.8 years, the local control rate was 80% after proton therapy. Fifty percent of patients developed late toxicity, including 15% of patients with grade 3 and higher keratitis. Grade 3 or higher brain necrosis was observed in 15% of the patients. Holliday et al., in a retrospective study of 20 patients with a variety of tumors of the orbit and ocular adnexa, reported a local control rate of 100% after proton beam therapy at MD Anderson [14]. Only three patients in this study had skin cancer. The median follow-up of this study was only 27 months. Damico et al. reported a local control rate of 71% in 17 patients with peri-orbital cancer treated with proton beam therapy. Only two patients in this study had skin cancer. The median follow-up was 19.7 months [15]. As late effects of radiation commonly increase with time, long-term follow-up is crucial in this group of patients.

Our current study looked exclusively at patients with periocular skin cancer. It represents the only study that reports the long-term outcome of the use of protons in this patient population. Our current study is a retrospective study with a small number of patients; therefore, it has inherent biases. It should also be noted that proton therapy is a unique treatment modality that is not widely available, and its proper use relies on many years of experience. The findings of this study need to be confirmed with prospective multi-institutional trials with standardized ophthalmological assessment. Until this is studied prospectively, we must rely on high-quality retrospective series with long-term follow-up to inform our decisions.

## 5. Conclusions

Protons enable long-term tumor control with eye sparing in patients with locally advanced periocular skin cancers. For patients with advanced periocular cancers where Mohs surgery cannot be performed, ocular-sparing radiotherapy, such as proton therapy, should be offered as a treatment option in addition to enucleation or exenteration.

## Figures and Tables

**Table 1:** Patient, tumor, and treatment characteristics.

Characteristics	Number of Lesions (%)
**Age, years (range)**	76.5 (56–85)
**Median ECOG (range)**	1 (0–3)
**Gender**	
Male	3 (21)
Female	11 (79)
**Caucasian race**	14 (100)
**Diabetes**	1 (7)
**Hypertension**	11 (79)
**Smoking history**	5 (36)
**Tumor**	
Newly diagnosed	5 (36)
Recurrent cases	9 (64)
Median number of recurrences before protons (range)	1 (0–3)
**Primary site**	
Lower eyelid	6 (43)
Lateral canthus	2 (14)
Median canthus	6 (43)
**Tumor histology**	
Basal cell carcinoma	9 (64)
Squamous cell carcinoma	3 (22)
Sebaceous carcinoma	2 (14)
**Perineural**	3 (21)
**Orbital involvement**	13 (93)
**Extent of surgery**	
Gross total resection	2 (14)
Partial resection	10 (72)
Biopsy only	2 (14)
**Concurrent chemotherapy**	1 (7)
**Proton dose**	
Median GTV [Gy(RBE)] (range)	66.6 (66–70)
Median CTV [Gy(RBE)] (range)	60 (54–66)

Abbreviation: ECOG = Eastern Cooperative Oncology Group; GTV = Gross Tumor Volume; CTV = Clinical Target Volume; Gy(RBE) = Gray(Relative Biological Effectiveness).

## Data Availability

The original contributions presented in this study are included in the article. Further inquiries can be directed to the corresponding author.
